# Heard in the Corridor

**Published:** 1920-09-11

**Authors:** 


### Heard in the Corridor.

IT is well known that hospital secretaries, like other members of a hospital staff, are ill-paid, and perhaps that may account for the following episode:

The secretary of a hospital was standing in the front corridor of the hospital when he was approached by a young hall-maid (most secretaries will understand the peculiar type of domestic servant one has to put up with nowadays) who said to him "that the gentleman in the white coat wot lives round the corner (meaning the Resident Medical Officer) has told me that his fire has gone out and it not being half cold he wants you to light it at once." The secretary stirred the ashes, whereupon the fire spread rapidly to the three persons, and the R.M.O. had some difficulty to put it out.

